# Development and validation of a novel scoring model for predicting underlying intracranial atherosclerosis prior to endovascular treatment in acute posterior circulation large-vessel occlusion

**DOI:** 10.3389/fneur.2025.1609682

**Published:** 2025-07-23

**Authors:** Guoyi Peng, Chuming Huang, Jiaqi Huang, Qiuhui Shi, Wei Xu, Shiwei Luo, Jiong Yang, Shouxing Wang, Qiao Wu, Chuwei Cai, Hao Long

**Affiliations:** ^1^Department of Neurosurgery, Nanfang Hospital, Southern Medical University, Guangzhou, China; ^2^Institute of Brain Diseases, Nanfang Hospital, Southern Medical University, Guangzhou, China; ^3^Department of Neurosurgery, Shantou Central Hospital, Shantou, China; ^4^Department of Neurology, Shantou Central Hospital, Shantou, China; ^5^Department of Nutrition, First Affiliated Hospital, Shantou University Medical College, Shantou, China; ^6^Department of Neurosurgery, Haifeng Hospital, Shanwei, China; ^7^Department of Neurology, Chaozhou Central Hospital, Chaozhou, China; ^8^Department of Neurology, Jieyang People Hospital, Jieyang, China; ^9^Department of Neurology, Shanwei Second People Hospital, Shanwei, China; ^10^Department of Neurology, Dafeng Hospital, Shantou, China

**Keywords:** vertebrobasilar artery occlusion, endovascular treatment, atherosclerosis, stenosis, stroke

## Abstract

**Background and objective:**

Determining the cause of occlusion prior to endovascular treatment (EVT) for acute ischemic stroke caused by large-vessel occlusion (LVO) is helpful for developing a procedure strategy. The aim of this study was to develop and validate a novel scoring model to predict intracranial atherosclerosis-related large-vessel occlusion (ICAS-LVO) in patients with acute vertebrobasilar artery occlusion.

**Methods:**

The derivation cohort comprised 170 patients who received EVT between January 2018 and June 2024 at multiple centers. The validation cohort comprised 63 patients treated at other centers between June 2019 and December 2024. ICAS-LVO was defined as stenosis >70% or >50% accompanied by hemodynamic disturbances. The relationships between risk factors and ICAS-LVO were assessed via univariate and multivariate logistic regression analyses. The risk factors were used to develop a predictive model. The accuracy of the predictive model was then assessed by the area under the receiver operating characteristic curve (AUROC) in both the derivation and validation cohorts.

**Results:**

ICAS-LVO was found in 106 (62.4%) and 41 (65.1%) patients in the derivation and validation cohorts, respectively. After binary logistic regression, 5 items were associated with ICAS-LVO, including male sex [odds ratio (OR), 1.05; 95% confidence interval (CI), 1.02–8.09] (*p* = 0.047), history of hypertension [OR, 1.62; 95% CI, 1.72–14.91] (*p* = 0.003), atrial fibrillation (AF) [OR, 0.08; 95% CI, 0.03–0.25] (*p* = 0.001), mydriasis [OR, 0.22; 95% CI, 0.07–0.71] (*p* < 0.011) and terminal basilar artery involvement [OR, 0.12; 95% CI, 0.05–0.30] (*p* = 0.001). A scoring model was created on the basis of the *β* coefficients of these 5 factors, which demonstrated good calibration ability (Hosmer–Lemeshow test, *p* = 0.814) and discrimination power (AUROC: 0.898; 95% CI, 0.847–0.950). In the validation cohort, the AUROC, sensitivity and specificity were 0.895 (95% CI, 0.813–0.977), 85.4 and 81.8%, respectively.

**Conclusion:**

The scoring model, which was constructed on the basis of male sex, history of hypertension, AF, mydriasis and terminal basilar artery involvement, is a simple and accurate tool for predicting ICAS-LVO before EVT.

## Introduction

Endovascular treatment (EVT) has been proven to be safe and effective for acute ischemic stroke caused by acute posterior circulation large-vessel occlusion (LVO) (1, 2). However, more than 55% of patients have unfavorable outcomes after EVT (1–3). Fewer operations and shorter recanalization times are crucial for improving patient outcomes in the treatment of posterior circulation LVO (4). The clear identification of the underlying cause prior to EVT is particularly crucial in achieving this objective. Different occlusive etiologies lead to differences in recanalization strategies and device choices (5). In treating posterior circulation embolism LVO, the use of current devices, such as contact aspirators or stent retrievers, for recanalization results in satisfactory outcomes (6). However, the treatment of posterior circulation intracranial atherosclerosis-related large-vessel occlusion (ICAS-LVO) lesions appears to be more complicated (7). It has been reported that in cases of ICAS lesions, primary thrombectomy by a stent retriever over contact aspiration results in higher initial effective reperfusion rates (8). Furthermore, EVT involving only the use of a stent retriever may be insufficient in patients with ICAS-LVO, especially in the posterior circulation (8–11). This is because posterior circulation ICAS-LVO is usually accompanied by severe residual stenosis, which is associated with a higher rate of reocclusion (12). Research has shown that a relatively large proportion of posterior circulation ICAS-LVO lesions require further angioplasty and stenting or more intensive antiplatelet treatment (administration of tirofiban via intravenous or combined intravenous/intra-arterial routes before the first thrombectomy to reduce the risk of vascular reocclusion) (12–14). Prompt identification of the type of occlusion is helpful for selecting the optimal device and ensuring a smooth EVT procedure for treating posterior circulation LVO.

Many reports have revealed that higher baseline National Institutes of Health Stroke Scale (NIHSS) scores, collateral circulation, history of hypertension, hypercholesterolemia and atrial fibrillation (AF) are closely related to ICAS-LVO (15–17). Several scales have been used to predict the cause of anterior circulation LVO (16, 17), but they cannot be directly applied to posterior circulation LVO patients. A three-item scale including history of hypertension, AF and elevated baseline serum glucose levels was developed to predict posterior circulation ICAS-LVO, but the scale was likely to be insufficient because of the factors included (18). Additionally, the small sample size and low predictive performance also limit its widespread promotion (18). Hence, few reliable methods for predicting ICAS-LVO in the posterior circulation exist.

Thus, we conducted a multicenter, retrospective consecutive cohort study to systematically analyze the potential risk factors and efficacy of the model for predicting posterior circulation ICAS-LVO before EVT. Furthermore, a cohort from another center was used to verify the predictive model.

## Patient selection

This study was approved by the Ethics Committees of Shantou Central Hospital, Chaozhou Central Hospital, Jieyang People Hospital, Shanwei Second People Hospital, Haifeng Hospital, and Dafeng Hospital (no. 2022039). More than 30 EVT procedures are performed annually in all of these centers. This retrospective observational study was conducted in these hospitals between January 2018 and December 2024 (clinical trial number: not applicable). Written consent for patient information to be stored in the hospital database and used for research purposes was given by the patients or their families.

The following criteria were used for EVT (2, 18): patients who were diagnosed with vertebral and/or basilar artery occlusion by computed tomography angiography (CTA) or digital subtraction angiography (DSA); age ≥18 years; and treatment within 24 h of symptom onset or more than 24 h after symptom progression (defined as an NIHSS score ≥4 before treatment) and were considered to have a favorable benefit–risk ratio. We excluded patients with both posterior and anterior circulation cerebral artery occlusions; patients with occlusion in the posterior cerebral artery; patients with conditions such as dissection, vasculitis or an unclear pathogenesis; and patients with a modified Rankin scale (mRS) score > 2 before onset.

### Data collection

LVO-ICAS was defined according to the following criteria (17, 18): (a) severe (>70%) stenosis; (b) moderate stenosis (>50%) with significant distal flow impairment at the occluded segment when successful reperfusion was achieved or transient visualization of eccentric plaque contour or recurrent reocclusion when reperfusion was unsuccessful; and (c) intracranial atherosclerotic stenosis with large-vessel occlusion independently diagnosed by two experienced neurologists (GYP and another neurologist at each center). Cohen’s kappa coefficient was used to assess the consistency of the etiology classification by two independent raters. Any discrepancies in their assessments were resolved through consultation with an experienced interventionalist (CWC). In cases where a consensus could not be reached following discussion among the three specialists, the etiology was deemed unclear, and the patient was subsequently excluded from the study.

The NIHSS score was used to evaluate the severity of each patient’s conditions upon admission, and the results of head CT, routine blood examination and emergency electrocardiography were obtained in a timely manner. The posterior circulation Alberta Stroke Program early computed tomography score (pc-ASPECTS) was recorded according to the admission CT. Patients receiving intravenous thrombolytic therapy are recommended for green channel stroke treatment. In cases of residual stenosis of the vessels or stent implantation, antiplatelet drugs were used according to the patient’s condition during and after EVT.

We used the NIHSS score and the pc-ASPECTS to assess the severity of stroke and cerebral ischemic extension in the acute phase, respectively. A modified thrombolysis in cerebral infarction (mTICI) score ≥2b was considered to indicate successful recanalization (1). The basilar artery on computed tomography angiography (BATMAN) score was calculated on the basis of CTA or DSA features (19). Patient outcomes were assessed according to the modified Rankin scale (mRS) score on day 90 after EVT. An mRS score of 0–3 was considered to indicate good recovery, and an mRS score of 4–6 was considered to indicate poor recovery.

The following variables were recorded: age; sex; baseline NIHSS score; history of hypertension; diabetes mellitus; AF, including previous history, emergency electrocardiography; intravenous thrombolysis; mean arterial pressure (MAP); neutrophil–lymphocyte ratio; mydriasis before EVT, including bilateral anisocoria and bilateral pupil dilation; fetal posterior cerebral artery (fPCA) variant; baseline pc-ASPECTS; BATMAN score; terminal basilar artery involvement; time from symptom onset to groin puncture; EVT time; occlusion etiology; symptomatic intracranial hemorrhage (SICH); and patient outcomes.

### Statistical analysis

After data collection, a database was established for analysis using SPSS 29.0 statistical software and R software (version 4.3.1). For the general clinical data of patients, if the measurement data were normally distributed, a two-sample t test was used; otherwise, a two-sample nonparametric Mann–Whitney U test was used. Count data were tested using the chi-square or exact probability method. Univariate logistic regression analysis was used to initially determine whether sex, history of hypertension, diabetes, AF, BATMAN score, time from symptom onset to groin puncture, and terminal basilar artery involvement were associated with occlusion. Factors with a *p* value < 0.1 in the above univariate analysis were introduced into multivariate logistic stepwise regression analysis to determine the independent risk factors affecting the prognosis of patients with posterior circulation LVO. The Youden index (specificity + sensitivity −1) was maximized, and receiver operating characteristic (ROC) curve analysis was used to determine the best cutoff value for each independent risk factor. On the basis of the best cutoff value, the risk factors were converted into binary variables. A score was derived based on the results of the multivariate regression and the *β* coefficients of the factors (1 for male sex, 2 for hypertension, −1 for mydriasis, −2 for AF and −2 for terminal basilar artery involvement). The Hosmer–Lemeshow test was used to test the calibration ability. The sensitivity, specificity and prediction efficiency of the optimal scoring model were subsequently verified via receiver operating characteristic (ROC) curves. The results are expressed as the means (standard deviations), medians (interquartile spacings), or odds ratios (95% confidence intervals). In this study, a *p* value < 0.05 was considered to indicate a statistically significant difference.

## Results

### General characteristics

A total of 196 posterior circulation LVO patients in the developed cohort received EVT between January 2018 and June 2024 at 3 centers (Shantou Central Hospital, Chaozhou Central Hospital, and Jieyang People Hospital). Twenty-sixty patients were excluded for the following reasons: posterior cerebral artery occlusion (*n* = 5), dissection (*n* = 8), undetermined etiology (*n* = 3) (2 for ICAS or dissection and 1 for ICAS or embolism), baseline mRS > 2 (*n* = 2), incomplete DSA data or documentation (*n* = 4), and other (*n* = 4). The validation cohort comprised 63 patients treated at Shanwei Second People’s Hospital, Haifeng Hospital, and Dafeng Hospital between June 2019 and June 2024 and Shantou Central Hospital between July 2024 and December 2024. Ultimately, 170 patients were included in the developed cohort. There were 130 men and 40 women, and the mean age was 64.59 years (range: 35–91). A total of 134 patients had a history of hypertension, and 64 had diabetes mellitus. A total of 44 patients presented with a history of AF or emergency electrocardiogram features indicating AF. The baseline median NIHSS score was 17 (IQR, 13–19), the baseline pc-ASPECTS was 8 (IQR, 7–10), and the BATMAN score was 8 (IQR, 7–9). Seventeen of the 170 patients received intravenous thrombolysis. The MAP was 111.55 mmHg (SD, 18.78). The time from symptom onset to groin puncture and the duration of EVT was 537.88 (SD, 457.36) and 122.62 (SD, 57.12) minutes, respectively. Among the 170 patients, 23 patients received EVT more than 24 h after presentation. A total of 23 patients presented with tandem lesions. Among the 170 patients, 155 (91.2%) experienced successful recanalization (mTICI ≥ 2b). Seventy-two (42.4%) patients had good functional outcomes (mRS 0–3). Finally, 106 of the 170 patients (62.4%) in the derivation cohort and 41 of the 63 patients (65.1%) in the validation cohort were diagnosed with ICAS-LVO. Fourteen patients showed inconsistent results during the initial etiology assessment. Cohen’s kappa analysis revealed an interrater agreement of *κ* = 0.910 (*p* < 0.001) between the researchers.

Most patients with ICAS-LVO were male and had a history of hypertension, no AF, a long puncture time, a longer EVT time, no mydriasis before EVT, and a higher baseline pc-ASPECTS as well as terminal basilar artery involvement (Table 1). No significant differences were observed in terms of age, diabetes mellitus status, intravenous thrombosis incidence, MAP, presence of fPCA, SICH rate, mTICI, or 90-day mRS score. The baseline characteristics of the patients with ICAS-LVO in both the development and validation groups are compared in Table 1.

**Table 1:** Comparison of baseline demographic, clinical, and radiological characteristics between the ICAS-LVO and embolism LVO groups.

Characteristics	Derivation cohort (*n* = 170)			Validation cohort (*n* = 63)		
	ICAS-LVO (*n* = 106)	EM-LVO (*n* = 64)	*P*-value	ICAS-LVO (*n* = 41)	EM-LVO (*n* = 22)	*P*-value
Male (%)	89 (84.0)	41 (64.1)	0.003^a^	32 (78.0)	12 (54.5)	0.053^a^
Age (SD)	63.8 (9.3)	65.9 (12.8)	0.223^b^	61.9 (10.9)	65.7 (12.4)	0.216^b^
Hypertension (%)	94 (88.7)	40 (62.5)	0.001^a^	34 (82.9)	14 (63.6)	0.087^a^
Diabetes Mellitus (%)	45 (42.5)	19 (29.7)	0.096^a^	15 (36.6)	7 (31.8)	0.705^a^
Intravenous (%)	13 (12.3)	4 (6.3)	0.205^a^	3 (7.3)	2 (9.1)	1.000^d^
MAP (SD)	113.5 (19.3)	108.3 (17.6)	0.081^b^	113.2 (17.5)	109.0 (16.4)	0.360^b^
EVT Time (SD)	137.7 (52.3)	97.6 (56.4)	0.001^b^	167.5 (75.7)	114.1 (70.9)	0.009^c^
Time to Puncture (SD)	615.4 (534.1)	409.6 (242.7)	0.004^b^	459.9 (264.1)	318.9 (197.3)	0.034^c^
NLR (SD)	9.2 (8.0)	8.7 (7.9)	0.727^a^	7.4 (5.06)	6.4 (4.64)	0.451^d^
fPCA	23 (21.7)	15 (23.4)	0.792^a^	10 (24.4)	1 (4.5)	0.079^d^
Mydriasis	8 (7.5)	24 (37.5)	0.001^d^	6 (14.6)	5 (22.7)	0.494^d^
TBA involvement	27 (25.5)	53 (82.3)	0.001^d^	12 (29.3)	18 (81.8)	0.001^a^
AF (%)	9 (8.5)	35 (54.7)	0.001^a^	3 (7.3)	10 (45.5)	0.001^d^
NIHSS Score (IQR)	21 (13.75–27)	24 (17–27)	0.288^c^	16 (7.5–28)	23 (12.75–31.25)	0.083^c^
BATMAN (IQR)	8 (7–9)	7 (7–8)	0.001^c^	9 (7–9)	7 (6–9)	0.017^c^
ASPECTS (IQR)	8 (7–10)	8 (6–9)	0.145^c^	8 (7.25–10)	9 (8–10)	0.207^c^
mTICI <2b (%)	96 (90.6)	59 (92.2)	0.718^a^	38 (92.7)	21 (95.5)	1.000^d^
SICH	7 (6.6)	2 (3.1)	0.486^d^	2 (4.9)	1 (4.5)	1.000^d^
90 Day mRS (>3)	54 (50.9)	44 (68.8)	0.065^a^	23 (56.1)	13 (59.1)	0.773^a^

Significant differences were observed in sex, hypertension, EVT time, time from symptom onset to groin puncture, mydriasis, baseline NIHSS score, terminal basilar artery involvement, AF, and BATMAN score. AF, atrial fibrillation; CI, confidence interval; NLR, Neutrophil-to-Lymphocyte Ratio; NIHSS, National Institutes of Health Stroke Scale; MAP, mean arterial pressure; BATMAN, Basilar Artery on Computed Tomography Angiography; mTICI, modified thrombolysis in cerebral infarction; OR, odds ratio; pc-ASPECTS, posterior Alberta Stroke Program Early Computed Tomography Score; PCA, posterior cerebral artery; SD, Standard deviation; SICH, Symptomatic intracerebral hemorrhage; TBA, terminal basilar artery. ^a, b, c, d^compared by using Chi-square test, Student’s t-test, Mann–Whitney U test, and Fisher’s exact test, respectively.

### Regression analysis

Univariate logistic regression revealed that the following risk factors were related to ICAS-LVO: male sex [OR, 2.94; 95% CI, 1.42–6.08] (*p* = 0.004); history of hypertension [OR, 4.70; 95% CI, 2.14–10.31] (*p* < 0.001); BATMAN score [OR, 1.30; 95% CI, 1.04–1.64; for each score] (*p* ≤ 0.023); AF [OR, 0.08; 95% CI, 0.03–0.18] (*p* < 0.001); mydriasis before EVT [OR, 0.14; 95% CI, 0.06–0.33] (*p* < 0.001); and terminal basilar artery involvement [OR, 0.71; 95% CI, 0.03–0.16] (*p* < 0.001) (Table 2). All of those factors were included in the multivariate logistic regression analysis, and the results indicated that male sex [OR, 2.87; 95% CI, 1.02–8.09] (*p* = 0.047), history of hypertension [OR, 5.06; 95% CI, 1.72–14.91] (*p =* 0.003), AF [OR, 0.08; 95% CI, 0.03–0.25] (*p* < 0.001), mydriasis before EVT [OR, 0.22; 95% CI, 0.07–0.71] (*p* < 0.011) and terminal basilar artery involvement [OR, 0.12; 95% CI, 0.05–0.30] (*p* < 0.001) were independent predictors of ICAS-LVO (Table 3). The results in the validation cohort were similar (Table 3).

**Table 2:** Univariate regression analysis of predictors for ICAS-LVO.

Variable	Derivation cohort (*n* = 170)			Validation cohort (*n* = 63)		
	OR	95% CI	*P* -value	OR	95% CI	*P* -value
Male	2.94	1.42–6.08	0.004	2.96	0.97–9.07	0.057
Age	0.98	0.96–1.01	0.223	0.97	0.92–1.02	0.216
Hypetension	4.70	2.14–10.31	0.001	2.78	0.84–9.12	0.093
Diabetes Mellitus	1.75	0.90–3.38	0.098	1.24	0.41–3.71	0.705
Intravenous hrombolysis	2.10	0.65–6.73	0.214	0.79	0.12–5.12	0.804
MAP	1.02	1.00–1.03	0.083	1.02	0.98–1.05	0.356
Time to puncture	1.00	1.00–1.00	0.006	1.00	0.74–1.01	0.043
NLR	1.00	0.97–1.05	0.726	1.05	0.93–1.17	0.444
fPCA	0.91	1.43–1.90	0.792	6.77	0.81–56.94	0.078
Mydriasis	0.14	0.06–0.33	0.001	0.58	0.16–2.18	0.423
TBA involvement	0.71	0.03–0.16	0.001	0.09	0.03–0.33	0.001
AF	0.08	0.03–0.18	0.001	0.10	0.02–0.40	0.001
NIHSS Score	0.98	0.94–1.02	0.275	0.96	0.92–1.01	0.121
BATMAN (IQR)	1.30	1.04–1.64	0.023	1.34	0.97–1.86	0.077
ASPECTS (IQR)	1.14	0.95–1.36	0.151	0.81	0.54–1.20	0.290

Univariate logistic regression revealed that male sex, history of hypertension, BATMAN score, AF, mydriasis and terminal basilar artery involvement were associated with ICAS-LVO. AF, atrial fibrillation; CI, confidence interval; NLR, Neutrophil-to-Lymphocyte Ratio; NIHSS, National Institutes of Health Stroke Scale; MAP, mean arterial pressure; BATMAN, Basilar Artery on Computed Tomography Angiography; mTICI, modified thrombolysis in cerebral infarction; OR, odds ratio; pc-ASPECTS, posterior Alberta Stroke Program Early Computed Tomography Score; PCA, posterior cerebral artery; TBA, terminal basilar artery.

**Table 3:** Multivariate regression analysis of predictors for ICAS-LVO.

Variable	Derivation cohort (*n* = 170)				Validation cohort (*n* = 63)			
	β	OR	95%CI	*P*-value	β	OR	95%CI	*P*-value
Male	1.05	2.87	1.02–8.09	0.047	2.92	18.52	2.58–132.74	0.004
Hypetension	1.62	5.06	1.72–14.91	0.003	2.68	14.57	1.90–111.62	0.010
AF	−2.48	0.08	0.03–0.25	0.001	−3.09	0.05	0.01–0.39	0.005
Mydriasis	−1.52	0.22	0.07–0.71	0.011	-	-	-	-
TBA involvement	−2.16	0.12	0.05–0.30	0.001	−3.34	0.04	0.01–0.25	0.001

The analysis revealed that male sex, history of hypertension, AF, mydriasis and terminal basilar artery involvement were associated with ICAS-LVO. AF, atrial fibrillation; OR, odds ratio; CI, confidence interval; NIHSS, National Institutes of Health Stroke Scale; TBA, terminal basilar artery.

### Establishment and use of the predictive scale

To facilitate clinical use, we replaced the nomogram with a scoring system. On the basis of the risk factors and their *β* coefficients, a score was derived to accurately predict ICAS-LVO. Since β coefficients are noninteger values that are challenging to memorize and inconvenient for clinical application, we sought to develop a simplified scoring model based on these coefficients to enhance memorability and clinical utility. We evaluated multiple clinical prediction models using various numerical scoring parameters. Our results demonstrated that the model achieved optimal predictive efficacy when assigning the following scores: 1 point for male sex and 0 points for female sex; 2 points for hypertension and 0 points for no hypertension; −2 points for atrial fibrillation and 0 points for no atrial fibrillation; 1 point for mydriasis and 0 points for no mydriasis; and −2 points for terminal basilar artery involvement and 0 points for no terminal basilar artery involvement (range: −5 to 3) (Tables 3, 4).

**Table 4:** Components of the scoring model: 1 point for male sex and 0 points for female sex; 2 points for hypertension and 0 points for no hypertension; −2 points for atrial fibrillation and 0 points for no atrial fibrillation; 1 point for mydriasis and 0 points for no mydriasis; and −2 points for terminal basilar artery involvement and 0 points for no terminal basilar artery involvement (range: −5 to 3).

Items	Score
Male	
Yes	1
No	0
Hypertension	
Yes	2
No	0
AF	
Yes	−2
No	0
Mydriasis	
Yes	−1
No	0
TBA involved	
Yes	−2
No	0

**Figure 1:**
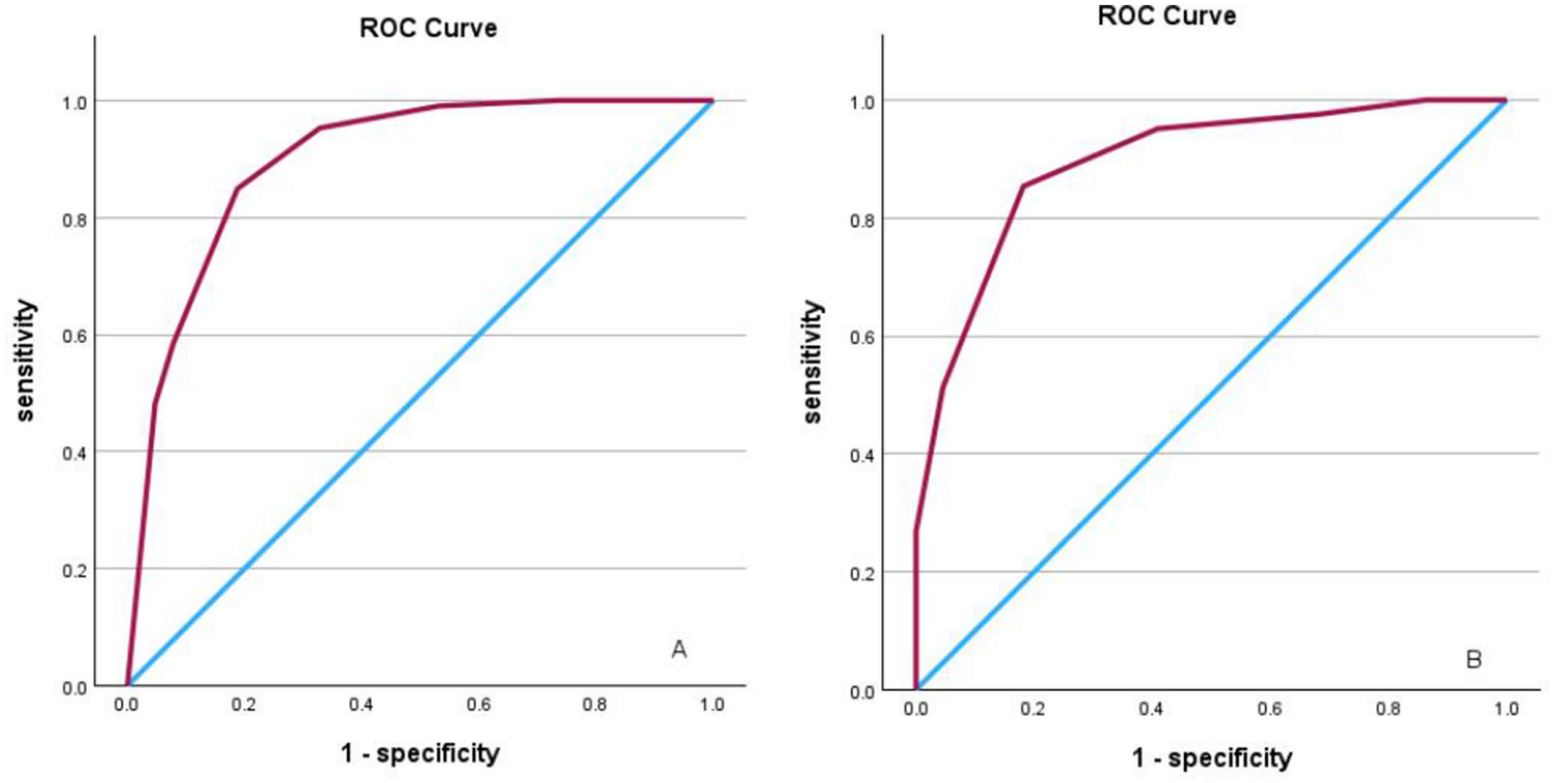
**(A)** ROC curve analysis illustrating scoring model discrimination in the derivation cohort, with an AUROC of 0.898 (95% CI, 0.847–0.950). The sensitivity and specificity were 84.9 and 81.2%, respectively. **(B)** ROC curve analysis illustrating scoring model discrimination in the validation cohort, with an AUROC of 0.895 (95% CI, 0.847–0.950); the sensitivity and specificity were 85.4 and 81.8%, respectively.

**Figure 2:**
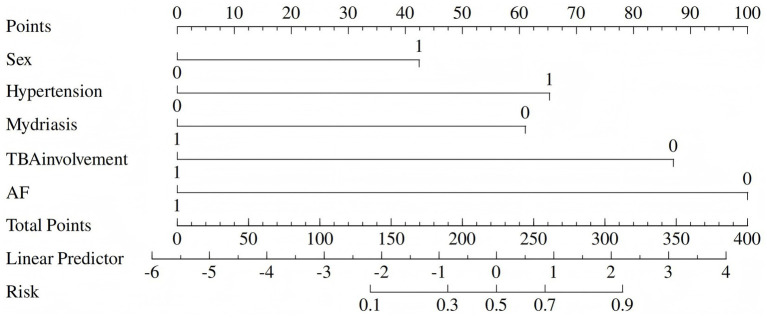
Nomogram for the incidence of ICAS-LVO.

The derived score model demonstrated satisfactory calibration, as indicated by the Hosmer–Lemeshow test (*p* = 0.814), and exhibited excellent discrimination, with an area under the curve of 0.898 (95% CI, 0.847–0.950). The optimal cutoff value identified for the ICAS-LVO scale was 0.5 point, with a sensitivity of 84.9% and specificity of 81.2% (as illustrated in Figure 1A). Those who had a score of −5 to −3 had a 0% probability of ICAS-LVO; those with a score of −2 to −1 had an approximately 20% probability of ICAS-LVO; and those with a score from 1–3 had an approximately 90% probability of ICAS-LVO (Table 5). The nomogram was used to assess the probability of ICAS-LVO (Figure 2).

**Table 5:** Incidence of ICAS-LVO before EVT on the basis of the derived score.

	Score (*n* = 170)								
	−5	−4	−3	−2	−1	0	1	2	3
	3	3	11	14	17	20	35	13	54
ICAS-LVO	0	0	0	1	4	11	28	11	51
Percentage (%)	0	0	0	7.1	23.5	55.0	80.0	84.6	94.4

Higher scores indicate a greater probability of ICAS lesions.

### Assessment of the predictive model in the validation cohort

In the validation cohort, comprising 63 patients, the scoring system demonstrated robust predictive efficacy, with an AUROC of 0.895 (95% CI, 0.847–0.950). Furthermore, the sensitivity and specificity were 85.4 and 81.8%, respectively (Figure 1B). This finding indicates the potential utility of the scoring system in clinical decision-making processes.

## Discussion

The purpose of this study was to develop an accurate and convenient model for predicting occlusion etiology in posterior circulation LVO patients. According to previous reports, cardiac embolism is the most common etiology of anterior circulation LVO (20). However, in posterior circulation LVO, the situation is reversed. In this study, we observed that 62.4% (106/170) of patients with posterior circulation LVO caused by ICAS underwent EVT, which is consistent with previous reports. Zha et al. reported that 43.2% of posterior circulation LVO cases were attributed to intracranial atherosclerosis (18). A study involving the largest sample of patients with posterior circulation LVO revealed that 64.9% of cases were due to intracranial arteriosclerosis, and another 27% were caused by cardioembolism (4). These studies revealed that ICAS-LVO is more common than embolism LVO is in patients with posterior circulation ischemia. The reasons underlying this phenomenon may be that the blood flow in the anterior circulation is greater than that in the posterior circulation, making it easier for clots to follow the blood flow into the anterior circulation rather than the posterior circulation.

Our results revealed that 5 factors, i.e., male sex, history of hypertension, no AF, no mydriasis, and no terminal basilar artery involvement, were associated with ICAS-LVO. Unexpectedly, the analysis did not reveal any relationship between the risk of ICAS-LVO and the NIHSS score, which is an important and familiar predictive factor of anterior circulation ICAS-LVO (16, 17), indicating similar severity for ICAS-LVO and embolism LVO of the posterior circulation. Interestingly, this study revealed that ICAS-LVO was more common in males than in females (5). A literature review revealed that in both the BAOCHE and ATTENTION trials—major studies on posterior circulation LVO—males predominated (73.2% in BAOCHE and 67.9% in ATTENTION) (1, 2). These findings align closely with our data (74.6%, 174/233), suggesting that posterior circulation LVO occurs more frequently in males. However, the phenomenon of ICAS-LVO being more common in males has been described in only a few studies because of small sample sizes, and this difference becomes more apparent as the number of patients increases (18). Not surprisingly, a history of hypertension was an independent risk factor for ICAS-LVO (16–18). Hypertension is the main cause of atherosclerosis (21). Many studies have revealed that a history of hypertension is strongly associated with ICAS-LVO (16–18), which is consistent with the findings of this study. In contrast, AF is a protective factor for ICAS-LVO. Patients with AF are prone to embolism (22), and many studies have revealed the relationship between AF and the risk of embolism LVO (16–18). In this study, patients with AF had 2-fold greater odds of ICAS-LVO than embolism LVO. Additionally, terminal basilar artery involvement has been confirmed to be a negative predictor of ICAS. Studies on the etiology of anterior circulation occlusion have revealed that ICAS-LVO is more common in the arterial trunk, whereas embolic-type occlusion is more frequently observed at arterial bifurcations (18, 23, 24). This is because thrombi often become lodged at bifurcations as they flow with the blood. Studies on the posterior circulation have revealed that ICAS is more common in the middle segment of the basilar artery (25). In this study, we selected terminal basilar artery involvement as a predictive factor because assessing whether the lesions are in the middle segment of the basilar artery is more challenging than evaluating whether the top of the basilar artery is involved. For example, in clinical practice, we often encounter cases where there is a significant thrombotic burden affecting the proximal–mid-segment or even the entire basilar artery, making it impossible to classify the specific lesion location. However, it is much easier to assess whether the terminal basilar artery is involved, thus making the model more user friendly. Moreover, caution is needed in certain special situations, such as when a patient has stenosis near the mid-segment of the basilar artery, where the thrombus may block the stenosed proximal end, potentially leading to embolic occlusion being misclassified as ICAS occlusion. This also confirms the difficulty of determining the type of lesion on the basis of a single factor, highlighting the need for more predictive factors for a comprehensive assessment. Preoperative pupil dilation is also negatively correlated with ICAS lesions. Previous studies have not revealed related findings, making this one of the more interesting discoveries. Since pupil observation is a mandatory part of our physical examination, we included it in our model. In other neurological diseases, pupil dilation is often closely associated with a poor prognosis (26). However, in this study, among patients with posterior circulation LVO who received timely EVT treatment, there was no significant difference in their prognosis regardless of postoperative pupil dilation. The mechanisms leading to pupil dilation in patients with brainstem infarction are diverse. Lesions in the dorsolateral pontine tegmentum may disrupt descending fibers from the midbrain Edinger–Westphal nucleus, leading to pupil dilation. In addition, the sympathetic nerve pathways in the brainstem being affected may also cause pupil changes. However, pupil dilation is most commonly observed in midbrain injury. Clinical studies have demonstrated ipsilateral mydriasis in most of midbrain infarction cases, primarily attributable to direct involvement of the oculomotor nuclear complex (27, 28). We speculate that embolic lesions are more likely to occur above the basilar artery, causing ischemia at the top of the basilar artery, which affects the nucleus of the oculomotor nerve, therefore causing oculomotor nerve paralysis and resulting in pupil dilation. Timely restoration of blood flow to the terminal basilar artery may prevent progression to midbrain death. This not only explains the relationship between pupil changes and the underlying cause but also clarifies that such pupil changes are not a prognostic factor and should not be used as a basis for denying EVT as a rescue measure for these patients.

Furthermore, to facilitate clinical application, we developed a novel etiological assessment scoring model based on these five factors instead of a normal nomogram model. This scoring model demonstrated superior assessment efficacy in the internal evaluation. Moreover, the 5 items, including history, physical examination and imaging results of the patients, are easy to access and evaluate in clinical settings. In the external validation cohorts from another multicenter study, the scale score also showed good calibration ability (Hosmer–Lemeshow test, *p* = 0.814) and high accuracy (AUROC, 0.895; 95% CI, 0.847–0.950). This model can assist interventionalists in accurately assessing the etiology of occlusion prior to procedures, enabling them to ascertain the optimal EVT strategy in advance. Moreover, patients with low NIHSS scores and onset times greater than 24 h were excluded from this study, which aligns with real-world clinical scenarios and highlights the widespread applicability of the model.

We found that those who had scores of −5 to −3 had a 0% probability of ICAS-LVO, and approximately 16% (5/31) of patients had scores of −2 and −1, indicating ICAS-LVO. Approximately 90% (90/102) of the patients with scores ranging from 1–3 were likely to be diagnosed with ICAS-LVO. Hence, in clinical practice, patients with a score of −5 to −2 exhibit near-certain radiographic confirmation of embolic occlusion. Given the established evidence that contact aspiration thrombectomy achieves superior recanalization outcomes for posterior circulation embolism LVO, an endovascular-first approach prioritizing suction-based techniques (e.g., ADAPT techniques) is recommended during procedural planning (29, 30). Additionally, this approach prevents iatrogenic endothelial damage by eliminating unnecessary traversal of microdevices (microwires/microcatheters) through the occluding lesion. In patients scoring −1 to 0 on our prediction model, where the probability of intracranial atherosclerosis (ICAS) versus embolic occlusion approaches equality (50% each), primary stent retrieval or contact aspiration represent equally reasonable initial strategies. If aspiration thrombectomy is attempted first, early transition to stent retriever deployment is recommended when unsuccessful first-pass recanalization occurs, thereby avoiding repeated aspiration attempts that may prolong ischemia. Nevertheless, we advocate prioritizing primary stent retrieval in this cohort. Real-time assessment of stent conformability during deployment provides critical diagnostic insight. Upon intraprocedural confirmation of an ICAS lesion, tirofiban should be administered immediately before thrombus extraction to mitigate the risk of reocclusion (13, 14). For patients with a score greater than 1, considering the high possibility of ICAS lesions, the use of stent retriever devices is recommended as the primary mechanical thrombectomy option (8). However, a significant proportion of posterior circulation ICAS lesions ultimately require adjunctive angioplasty or stent implantation. Although there is insufficient evidence demonstrating that rescue angioplasty improves patient outcomes, we still suggest that ICAS patients undergo timely angioplasty after a single failed thrombectomy attempt. This approach reduces iatrogenic injury caused by repeated maneuvers and expedites the procedure (7). However, in some cases, primary direct angioplasty or stenting over initial thrombectomy for ICAS-related occlusions may be reasonable. We suggest scoring the thrombotic load in terms of the first-pass effect prior to EVT (31). If the first-pass effect is achieved, the thrombotic load should be considered small, and direct balloon dilation or/and stent implantation should be considered (32–34); if the first-pass effect is not achieved, the thrombotic load should be considered heavy. Thus, it would be suitable to use a stent retriever to reduce the thrombotic load first. In addition, novel techniques such as the BASIS and BASIS-stent techniques should also be considered first-line choices (35). We believe that choosing the procedural strategy according to the characteristics of the lesions can reduce both the EVT time and the incidence of complications, resulting in improved patient outcomes.

There are several limitations to our study. First, the scoring model incorporates medical history, such as history of hypertension, history of AF and medical records related to the eyes, to rule out the influence on the judgment of the pupils, and an accurate medical history is required from patients without cognitive impairment or their families to ensure the reliability of the assessment. Second, as a retrospective study, potential information recall and selection bias cannot be ignored, even though we attempted to avoid such bias (e.g., ICAS was independently evaluated by multiple physicians). Third, several potential predictors, such as fetal PCA and terminal basilar artery involvement, were derived from CTA and/or DSA, and identifying all the factors before DSA would make the model more feasible. Importantly, the marked male predominance in our sample may constrain the generalizability of findings to female populations; consequently, the current model demonstrates higher clinical applicability for male patients and requires further validation in female-specific cohorts. Finally, the scoring model drawn from this study is limited by the small size of the validation cohort, and the data only came from several centers in southeastern China, which limits the wider applicability of the model.

## Conclusion

The novel scoring model, which was constructed on the basis of male sex, history of hypertension, AF, mydriasis and terminal basilar artery involvement, is a simple and accurate tool for predicting ICAS-LVO in patients with acute vertebrobasilar artery occlusion.

## Data Availability

The raw data supporting the conclusions of this article will be made available by the authors, without undue reservation.
